# Large-Scale Synthesis Route of TiO_2_ Nanomaterials with Controlled Morphologies Using Hydrothermal Method and TiO_2_ Aggregates as Precursor

**DOI:** 10.3390/nano11020365

**Published:** 2021-02-01

**Authors:** Wenpo Luo, Abdelhafed Taleb

**Affiliations:** 1 Institut de Recherche de Chimie Paris, PSL Research University Chimie ParisTech—CNRS, 75005 Paris, France; Wenpo.Luo@chimieparistech.psl.eu; 2 Sorbonne Université, 75231 Paris, France

**Keywords:** TiO_2_ nanoparticles, aggregates, morphologies

## Abstract

TiO_2_ of controlled morphologies have been successfully prepared hydrothermally using TiO_2_ aggregates of different sizes. Different techniques were used to characterize the prepared TiO_2_ powder such as XRD, XPS, FEGSEM, EDS, and HRTEM. It was illustrated that the prepared TiO_2_ powders are of high crystallinity with different morphologies such as nanobelt, nanourchin, and nanotube depending on the synthesis conditions of temperature, time, and additives. The mechanism behind the formation of prepared morphologies is proposed involving nanosheet intermediate formation. Furthermore, it was found that the nanoparticle properties were governed by those of TiO_2_ nanoparticles aggregate used as a precursor. For example, the size of prepared nanobelts was proven to be influenced by the aggregates size used as a precursor for the synthesis.

## 1. Introduction

Recently, tremendous efforts have been devoted to developing innovative strategies to synthesize nanomaterials with the desired morphologies and properties. Particularly the one-dimensional (1D) structure of TiO_2_ nanomaterials exhibits interesting properties compared to other TiO_2_ nanoparticles: it has lower carrier recombination rate and higher charge carrier mobility, thanks to the grain boundaries and junctions absence. In fact, the electron diffusion takes place through the junctions between nanoparticles, inducing slower charge transfer by several orders of magnitude [1]. In addition, it favors light scattering in the photoanode, which increases the light harvesting [2]. Among the studied morphologies and materials, semi conducting nanostructured materials such as nanowires, nanobelts, and nanotube have received particular attention, due to their use as photoanaode for potential applications in different areas such as photovoltaic [2], photo catalysis [3], gas sensing [4], and water photo-splitting [5].

Tuning the size and the morphology of materials is becoming a challenging goal in materials science. Over the past few years, various synthesis methods and protocols have been developed to control the semi-conducting nanomaterials morphology, including vapor–liquid–solid (VLS) [6], solution–liquid–solid (SLS) [7], template-based synthetic approaches [8,9], arc discharge [10], laser ablation [11], chemical vapor deposition [12], microwave [13,14], and sol–gel [15]. Among these synthesis methods, which mostly brought contamination to the synthesis products, the hydrothermal technique has been proven to be a simple and straightforward method using noncomplex apparatus, scalable for large production, with high chemical purity, allowing a large rang of nanomaterial sizes and morphologies [16,17]. Furthermore, the morphology of prepared TiO_2_ nanomaterials by using hydrothermal method was demonstrated to depend on the concentration of alkaline solution, the synthesis temperature and time, the material precursor used [17,18], additives, Pressure, pH, and the reaction medium [19,20,21,22,23,24,25].

Additionally, the hydrothermal method allows the control of the nanoparticles aggregation [26]. The most reported strategy to control the morphology of oxide nanomaterials is based on using organic surfactant, which adsorbs on a selected crystallographic plan of growing nucleus, leading to a change of its orientation and growth rate. This results in controlling the morphology of the obtained nanomaterial at the final growth stage [27,28]. Additionally, strategies based on aggregation/coalescence of nanomaterials were reported and demonstrated to be efficient in controlling the morphology of the final synthesized powder [29]. The exfoliation step was also reported to be a crucial step in the formation mechanism of prepared morphologies [29]. Most of the studies are based on nanomaterials aggregation/coalescence processes, and to the best of our knowledge, very few are based on exfoliation/aggregation/coalescence processes to explain synthesized morphologies. In the case of TiO_2_ nanomaterials, there is still a misunderstanding of the mechanism behind the formation of reported morphologies and particularly nanotube, nanobelt, and nanourchin. Some authors claimed that the Na_2_Ti_3_O_7_ nanosheets exfoliation step is the crucial step in the mechanism formation of different morphologies, whereas other authors stated that it is the dissolution of TiO_2_ nanoparticles into TiO_6_ octahedra, followed by Na_2_Ti_3_O_7_ nucleation and growth, forming a nanosheet in a later stage [29]. Furthermore, it is well accepted that different polymorphs of TiO_2_ nanomaterials are formed by different arrangements of TiO_6_ octahedra. In fact, the growth of anatase tetragonal polymorph proceeds through face sharing arrangements of TiO_6_ octahedras, whereas the rutile tetragonal phase growth takes place through edge-sharing arrangements. Furthermore, the Brookite phase is obtained by TiO_6_ octahedra assembly, sharing their edge and corner; whereas in Ti_2_O (B) (bronze) phase, Ti^4+^ ion form two distinct geometries with oxygen: octahedron in one case and a square pyramidal in the other. In addition, to homogeneous size and morphology, prepared TiO_2_ nanomaterials using hydrothermal method exhibit several characteristics such as high crystallinity, an accurate control of different crystallinity phases from anatase to rutile depending on the synthesis and annealing temperatures, and high specific surface [30]. It is well accepted that the anatase polymorph possesses a higher band gap energy (3.3 eV) than that of the rutile polymorph (3 eV).

In the present work, different morphologies of TiO_2_ have been successfully prepared hydrothermally using TiO_2_ aggregates made of TiO_2_ nanoparticles as a precursor. The mechanism behind the morphology control of prepared nanomaterials was discussed. It was found that the prepared TiO_2_ nanomaterials properties were governed by those of TiO_2_ nanoparticles aggregate. By controlling TiO_2_ nanoparticles and aggregate sizes, it has been demonstrated that it is possible to control the TiO_2_ nanobelt sizes.

## 2. Materials and Methods

### 2.1. Synthesis of TiO_2_ Nanoparticles

For the synthesis of TiO_2_ nanoparticles, titanium (IV) oxysulfate hydrate (TiOSO_4_, Sigma Aldrich, St. Louis, MO, USA) precursor was used. Furthermore, the synthesis of TiO_2_ aggregates has been performed using a hydrothermal synthesis technique. The TiOSO_4_ precursor solution was prepared by dissolving 6.4 g of TiOSO_4_ (2.5 M) in 16 mL of distilled water under constant stirring of 750 r/min and temperature of 45 °C for 2 h to get a clear solution. Then the solution of TiOSO_4_ was transferred into a Teflon-lined stainless-steel autoclave of 25 mL capacity. The heating rate was of 2.5 °C/min, and during the synthesis, the temperature was maintained at different temperatures of 100, 200 and 220 °C for 6 h depending on the aggregate size required. After this synthesis in autoclave, a white TiO_2_ powder was obtained and was washed six times in distilled water and two times in ethanol. Then the powder was dried overnight in the oven and annealed in air at temperature of 500 °C for 30 min with the heat rate of 5 °C/min. For nanourchin, nanotube, and nanobelt synthesis, 0.5 g powder of TiO_2_ aggregate was introduced in a Teflon-lined autoclave of 25 mL capacity. Then, the autoclave was filled with 10 M NaOH solution up to 80% of the autoclave capacity. During the synthesis, the temperature was maintained at different temperatures of 100, 150, and 220 °C with the heating rate of 2.5 °C/min and the synthesis time of 360, 180, and 15 min, depending on the required morphology. Afterwards, synthesis nanobelt particles are subjected to the washing and annealing protocols to obtain at the end of these processes: sodium titanate. The latter product was washed many times with diluted HCl solution to attain a pH value of 1. After that, the suspension was washed with distilled water several times to reach a pH value of 7. Finally, the obtained powder was dried overnight in the oven, and annealed in air at temperature of 500 °C for 30 min, with the heat rate of 5 °C/min.

All the chemicals are of analytical grade and used without further purification. The water used in all the experiments was purified by Milli Q System (Millipore, electric resistivity 18.2 MΩ.cm).

### 2.2. The Characterizations of TiO_2_ Films

The morphological investigations of the prepared films were achieved with a high-resolution Ultra 55 Zeiss FEG scanning electron microscope (FEGSEM) operating at an acceleration voltage of 10 kV and the high-resolution transmission electron microscope HRTEM using JEOL 2100 Plus microscope.

The crystalline structure of TiO_2_ was determined by an X-ray diffractometer (Siemens D5000 XRD unit) in 2θ range from 20° to 80° by 0.07°/s^−1^ increasing steps operating at 40 KV accelerating voltage and 40 mA current using Cu K*α* radiation source with λ = 1.5406 Å.

The chemical compositions of all the samples were determined by the FEGSEM using a Princeton Gamme-Tech PGT, USA, spirit energy dispersive spectrometry EDS system, and by X-ray photoelectron spectroscopy XPS realized with X-ray photoelectron spectroscopy (XPS), and for the measurements we used a Thermo K Alpha analyzer system equipped with an AL Kα X-ray source (hυ = 1486.6 eV; spot size 400 μm).

## 3. Results and Discussion

**Figure 1:**
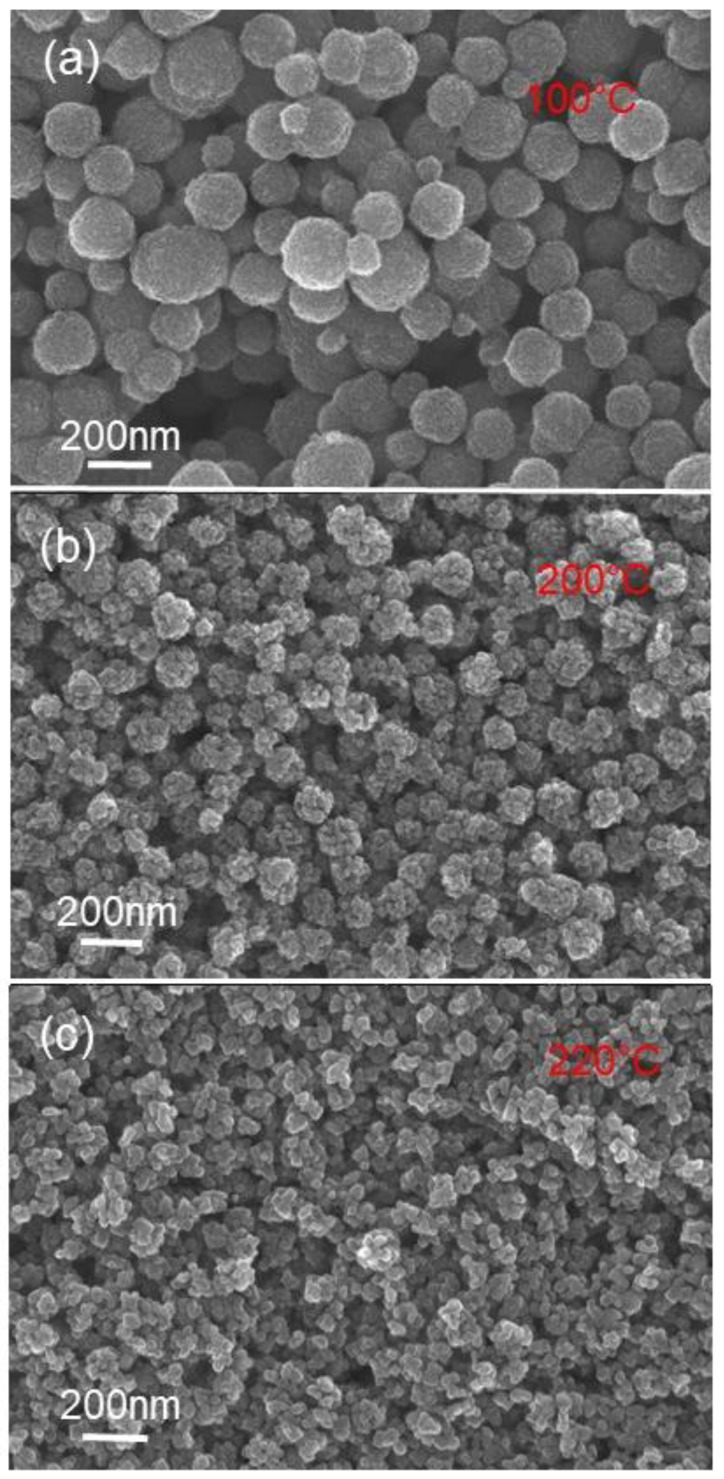
FEGSEM images of TiO_2_ aggregates obtained at different synthesis temperatures: (**a**) 100, (**b**) 200, and (**c**) 220 °C.

Various powders were prepared using the alkali hydrothermal synthesis method and varying synthesis temperatures and reaction times. To prepare these powders, TiO_2_ aggregates of spherical shape and different sizes were prepared and used as precursors. The FEG-SEM characterization of precursor powders are shown in Figure 1, and it can be observed that the sizes of spherical aggregates are ranging from 50 to 200 nm.

**Figure 2:**
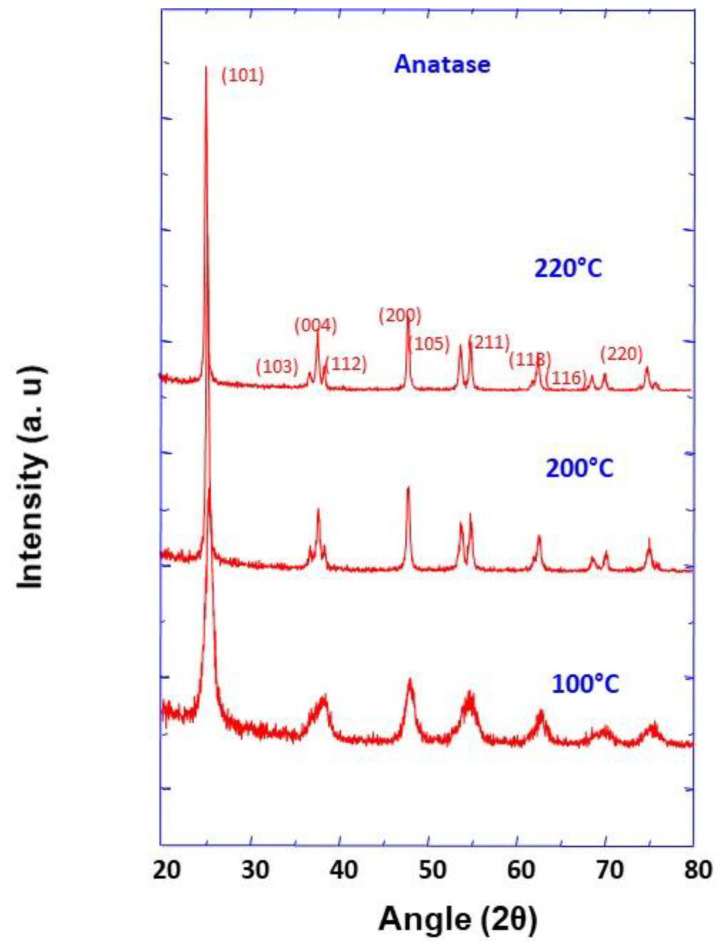
XRD pattern of TiO_2_ nanoparticle aggregates prepared at different synthesis temperatures as indicated.

The XRD method was used to characterize the crystalline phase of TiO_2_ aggregate precursors, and the obtained results are depicted in Figure 2. Several well-resolved peaks were observed and are all assigned to TiO_2_ anatase phase (JCPDS No. 21-1272), which is proof of the high purity of the prepared precursor powders. Additionally, Scherer analysis was used to calculate the average crystallite sizes at the half-maximum width of the intense peak corresponding to (101) crystallographic plane, and were found to be 9.8, 24.7, and 30.4 nm, for the synthesis temperatures of 100, 200, and 220 °C, respectively.

**Figure 3:**
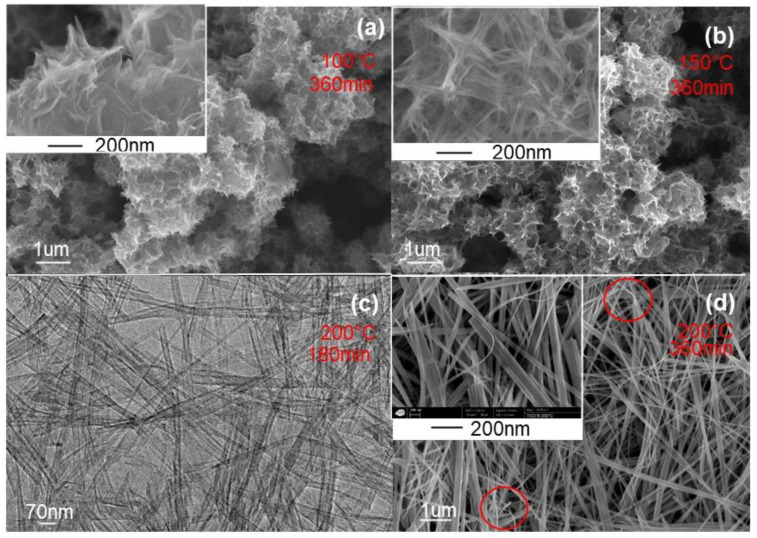
FEGSEM images of TiO_2_ nanoparticles with different morphologies obtained at different synthesis times and temperatures: (**a**) Nanourchin prepared at conditions of 100 °C and 360 min, (**b**) Nanourchin prepared at conditions of 150 °C and 360 min, (**c**) TEM image of Nanotube prepared at conditions of 200 °C and 180 min, and (**d**) Nanobelts prepared at conditions of 200 °C and 360 min.

White powders were obtained using TiO_2_ aggregate precursors whatever the preparation conditions, and their corresponding morphologies are depicted in Figure 3. As it can be observed, at the synthesis temperature of 100 °C, the morphology of the prepared powder is nanourchin-like with a stretched sheet-like network (Figure 3a), whereas at a temperature of 150 °C, the morphology is still nanourchin-like but with a more rolled nanosheet-like network (Figure 3b). From these experiments, it is clear that the temperature increase favors the nanosheet scrolling. This could be explained by the fact that the crystallization enhanced by the temperature increase tends to induce the microstructure to change into rolled nanosheet structure. In fact, to reduce the surface energy of rolled structure, nanosheets reduce the defects and the distortion energy [31]. At a higher temperature of about 200 °C, the FEGSEM characterization of prepared white powder is depicted in Figure 3c,d. It can be observed that TiO_2_ powder is of nanobelt-like and nanotube morphologies, with monodisperse size. The insert of Figure 3d shows a sticking of several distinguishable nanobelts along their axis direction, forming bundles of nanobelts as a building unit. It can also be observed that their thickness is homogeneous and it is of about 10 nm, their diameter is ranging from 50 to 100 nm with length of around 10 mm. In addition, the nanobelt surface is smooth at the magnification scale, and no contamination was observed. As indicated in Figure 3d, some curved nanobelts were observed, which gives an indication about their high elasticity. From the described experiments, it is clear that the synthesis temperature is an important parameter in the morphology control of TiO_2_ nanomaterials.

**Figure 4:**
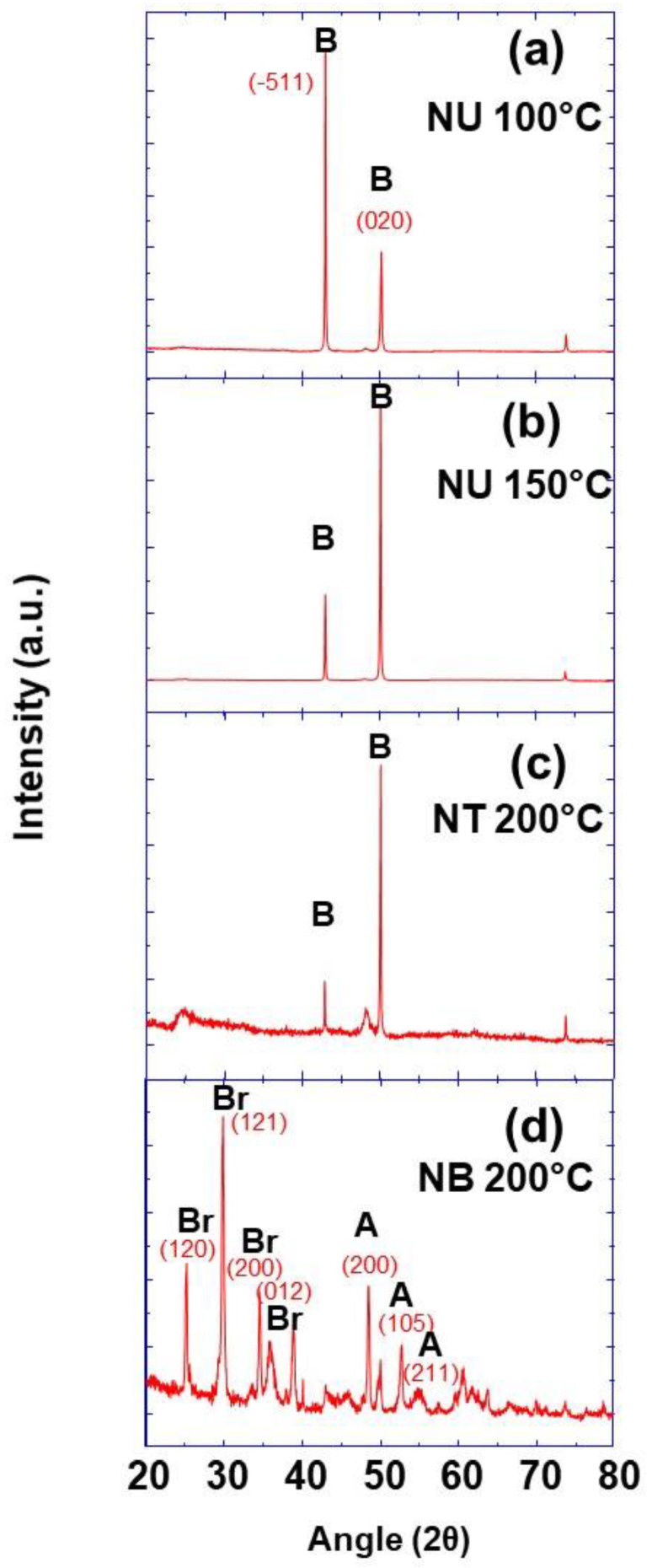
XRD pattern of TiO_2_ nanoparticles with different morphologies prepared at different synthesis temperatures as indicated, (**a**) nanoursin 100 °C, (**b**) nanoursin 150 °C, (**c**) nanotube 200 °C and (**d**) nanobelt 200 °C (Br: Brookite; A: Anatase; B: TiO_2_-B).

The crystalline structure and phase of prepared TiO_2_ nanobelt, nanotube, and nanourchin-like powders were studied by the X-ray diffraction method. The obtained XRD patterns are presented in Figure 4, and they show well-resolved peaks in the case of nanourchin and nanotube mophologies attributed to (-511) and (020) crystallographic planes of pure TiO_2_(B) phase (JCPDS No. 35-0088) (Figure 4a–c). In the case of TiO_2_ with nanobelt morphology, the observed XRD peaks indicates that the prepared powder is a mixture of anatase (JCPDS 21-1272) and brookite (JCPDS 29-1360) phases (Figure 4d).

**Figure 5:**
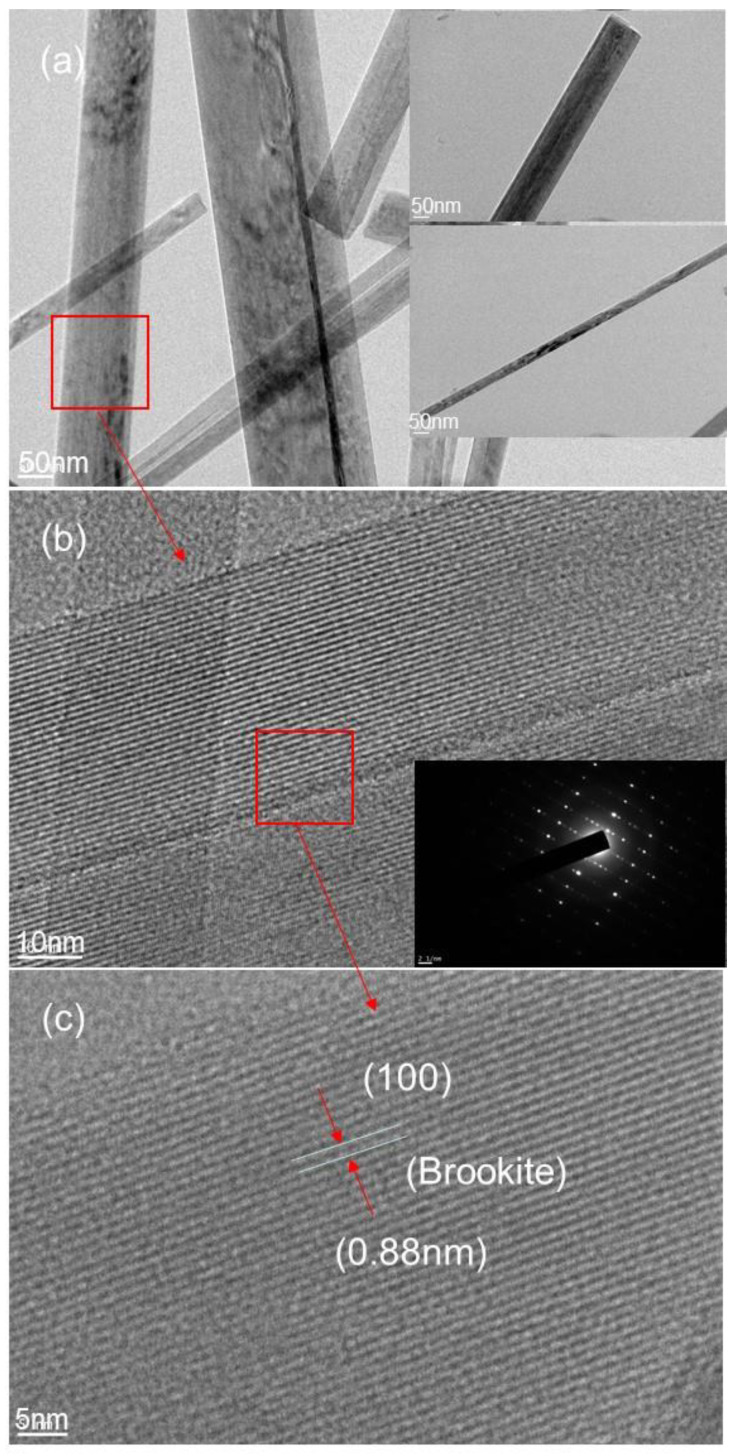
(**a**) TEM images of TiO_2_ nanobelt obtained at synthesis temperature of 200 °C a synthesis time of 6 h at different magnifications; (**b**) the corresponding HRTEM showing inter atomic crystallographic planes and the insert show the corresponding electron diffraction; (**c**) another magnification of TiO_2_ nanobelt.

Additionally, among all the peaks, the most intense is the one corresponding to (121) crystallographic plane of brookite. Further details of crystallinity are provided by HRTEM depicted in Figure 5, clearly well resolved lattice planes are shown, and the insert electron diffraction shows well resolved spots (Figure 5b). These spots are the signature that the individual nanobelt is a single crystal. The interplanar distance of about 0.88 nm measured from HRTEM image is assigned to (100) crystallographic plane of brookite, indicating that the growth takes place along the (100) crystallographic plane, which is in good agreement with the result from XRD experiments in terms of brookite formation.

**Figure 6:**
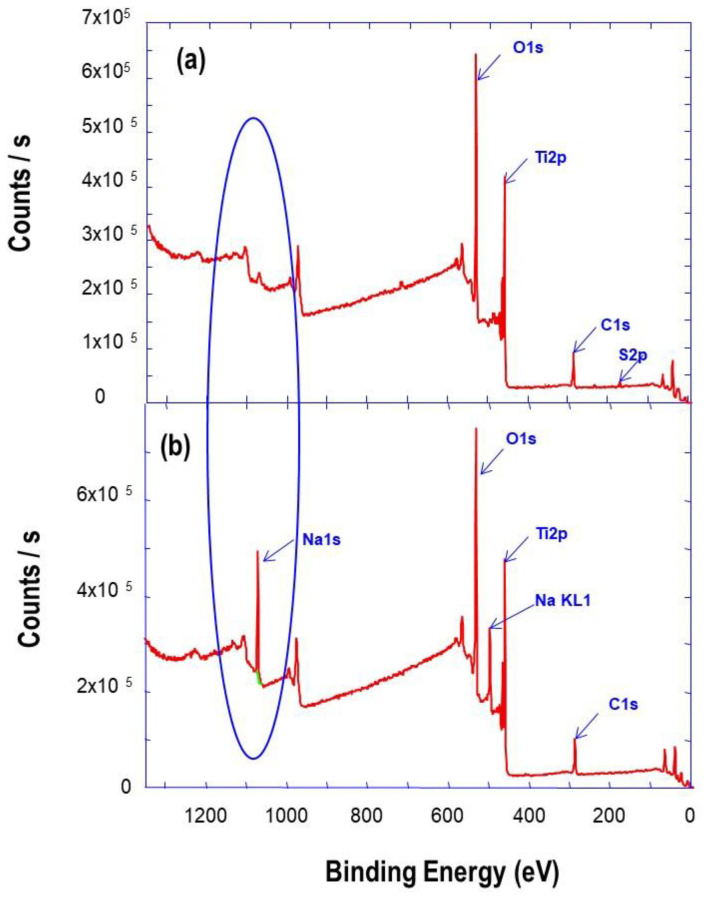
XPS survey spectra of prepared TiO_2_ (**a**) aggregate precursor (**b**) nanobelts after synthesis.

Furthermore, the chemical composition of the powder was provided by XPS analysis, and the obtained spectra are depicted in Figure 6. The XPS survey spectrum in Figure 6a of TiO_2_ aggregates precursor shows intense peaks corresponding to O1s and Ti2p core levels, and the very weak intensity of the peak corresponding to Na1s. However, the XPS survey spectrum corresponding to TiO_2_ nanobelt-like and nanourchin-like powders (Figure 6b) shows intense and well resolved peak, corresponding to the core level of Na1s, which is a signature of the formation of sodium titanate (Na_2_Ti_3_O_7_), in addition to those of O1s and Ti2p. It was reported that Na_2_Ti_3_O_7_ is constituted by corrugated strips of edge-sharing TiO_6_ octahedra [29]. The width of each strips is about three-octahedra, and they are connected through their corner to form stepped layers. Within the sticking layers, sodium cations are located at the positions between the layers.

**Figure 7:**
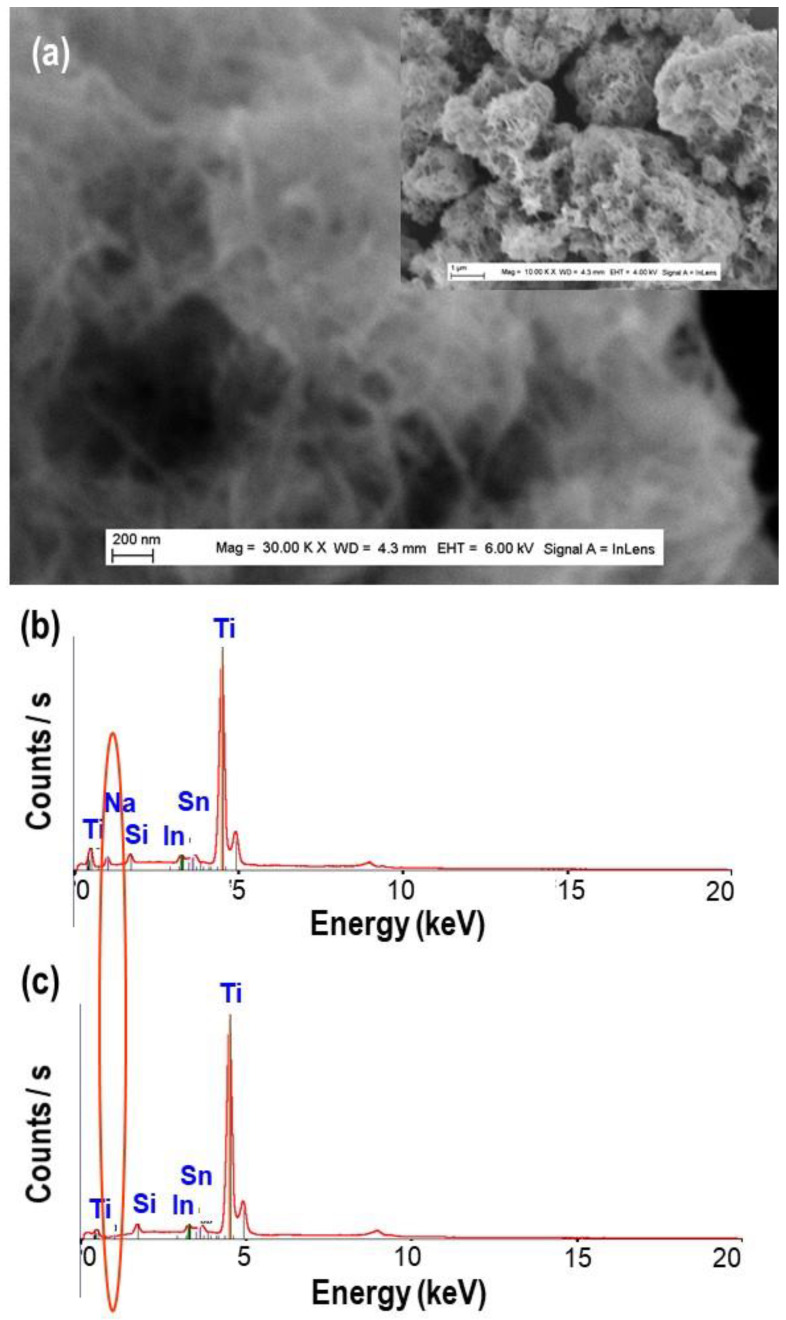
(**a**) FEGSEM images of TiO_2_ nanourchin obtained at synthesis temperatures of 150 °C, after washing and annealing; (**b**,**c**) the corresponding EDS spectrum obtained just after synthesis and after washing and annealing processes, respectively.

In Figure 7a, it is important to note that nanourchin-like nanoparticles show more enrolled nanosheet with more dense structure, as a consequence of the annealing process. The EDS analyses have been performed to determine the chemical composition of TiO_2_ nanoparticles, after just synthesis, or after washing and annealing processes. In Figure 7 the obtained EDS spectra are depicted; it should be noted that, on the EDS spectrum of TiO_2_ nanoparticles, after synthesis shows the presence of Na peak Figure 7b, whereas it is absent in the spectrum after the washing and annealing processes in Figure 7c. In fact, during the washing processes of Na_2_Ti_3_O_7_ by HCl, Na^+^ ions were exchanged by H^+^ ions. These results are a clear evidence of the important role played by Na^+^ ions in the formation of TiO_2_ nanobelts, nanotube, and nanourchin morphologies.

**Figure 8:**
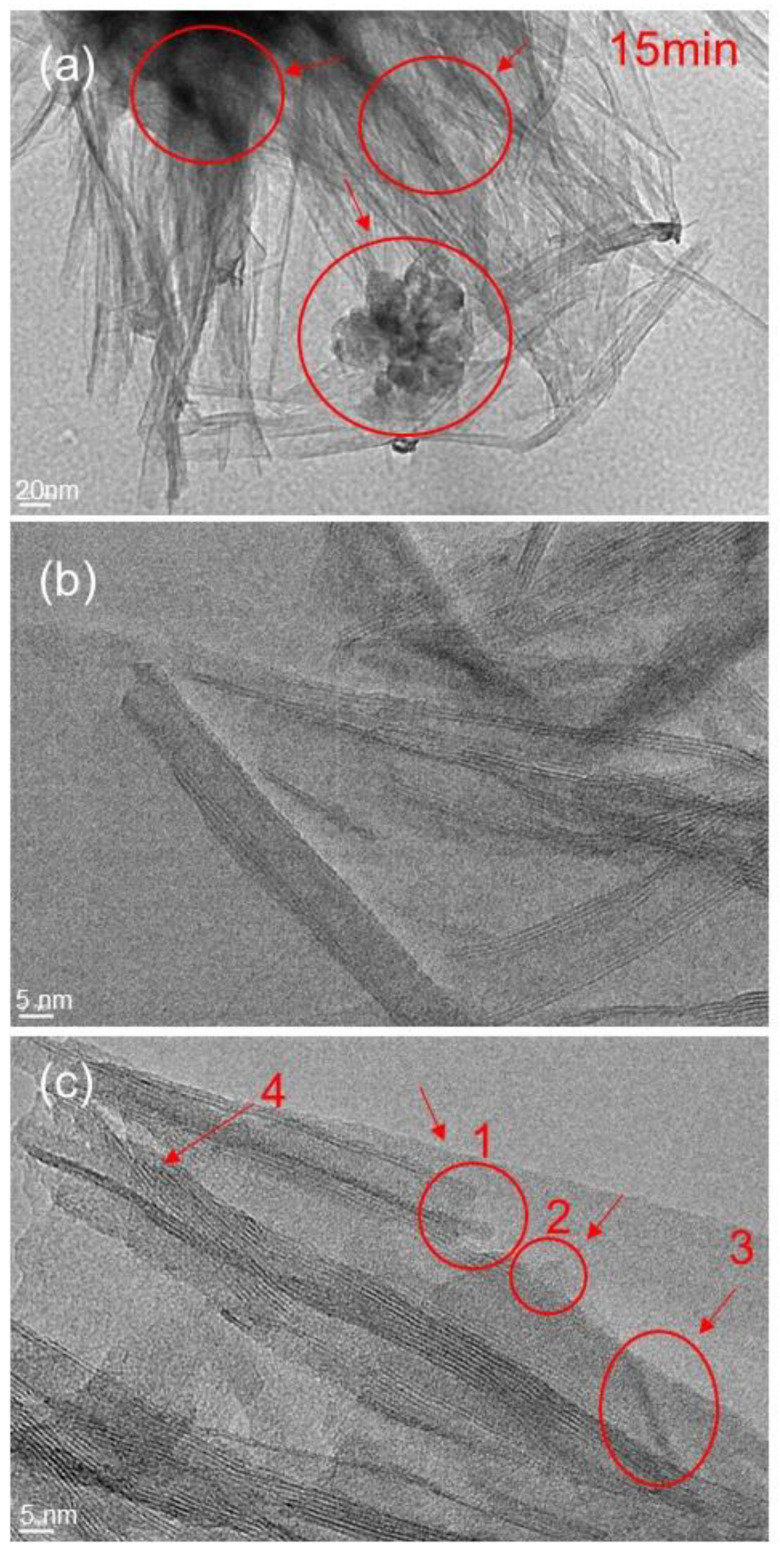
HRTEM images with different magnifications (**a**–**c**) of TiO_2_ nanotube prepared at synthesis temperature of 200 °C and synthesis time of 15 min.

The details of TiO_2_ nanobelt and nanotube formation mechanisms are further investigated by using high resolution transmission electron microscopy (HRTEM). The influence of hydrothermal reaction time on the morphology of prepared TiO_2_ nanomaterials is studied at 15, 180, and 360 min. At short reaction time of about 15 min, the morphology of prepared powder is mainly stretched nanosheet-like, with some minor rolled sheet. Closer analysis of prepared powder (Figure 8) shows different stages of the same formation mechanism.

**Figure 9:**
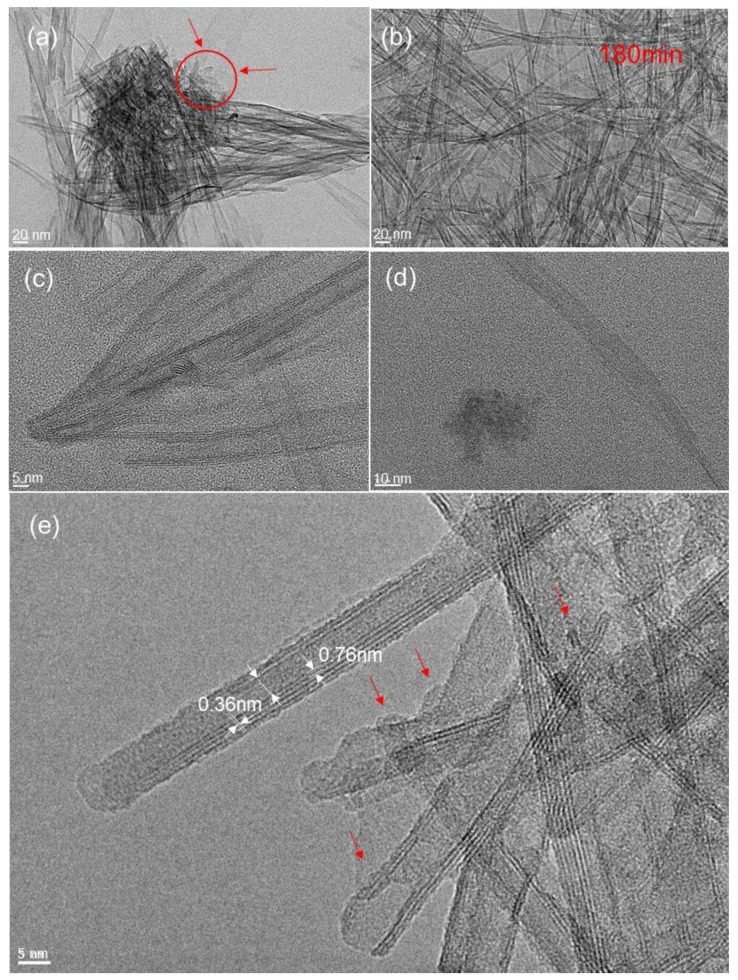
HRTEM images with different magnifications (**a**–**e**) of TiO_2_ nanotube prepared at synthesis temperature of 200 °C and synthesis time of 180 min.

In fact, the observed nucleation stage can be considered as an integrated growth process of nanobelt structure, from aggregates made of nanoparticles of about 20 nm diameter to nanobelt of several micrometers in length. Similar evolution was observed by other authors [4,29]. Thus, we may assume that the morphologies shown in Figure 8 and Figure 9 represent different stages of the nanobelt growth process.

**Figure 10:**
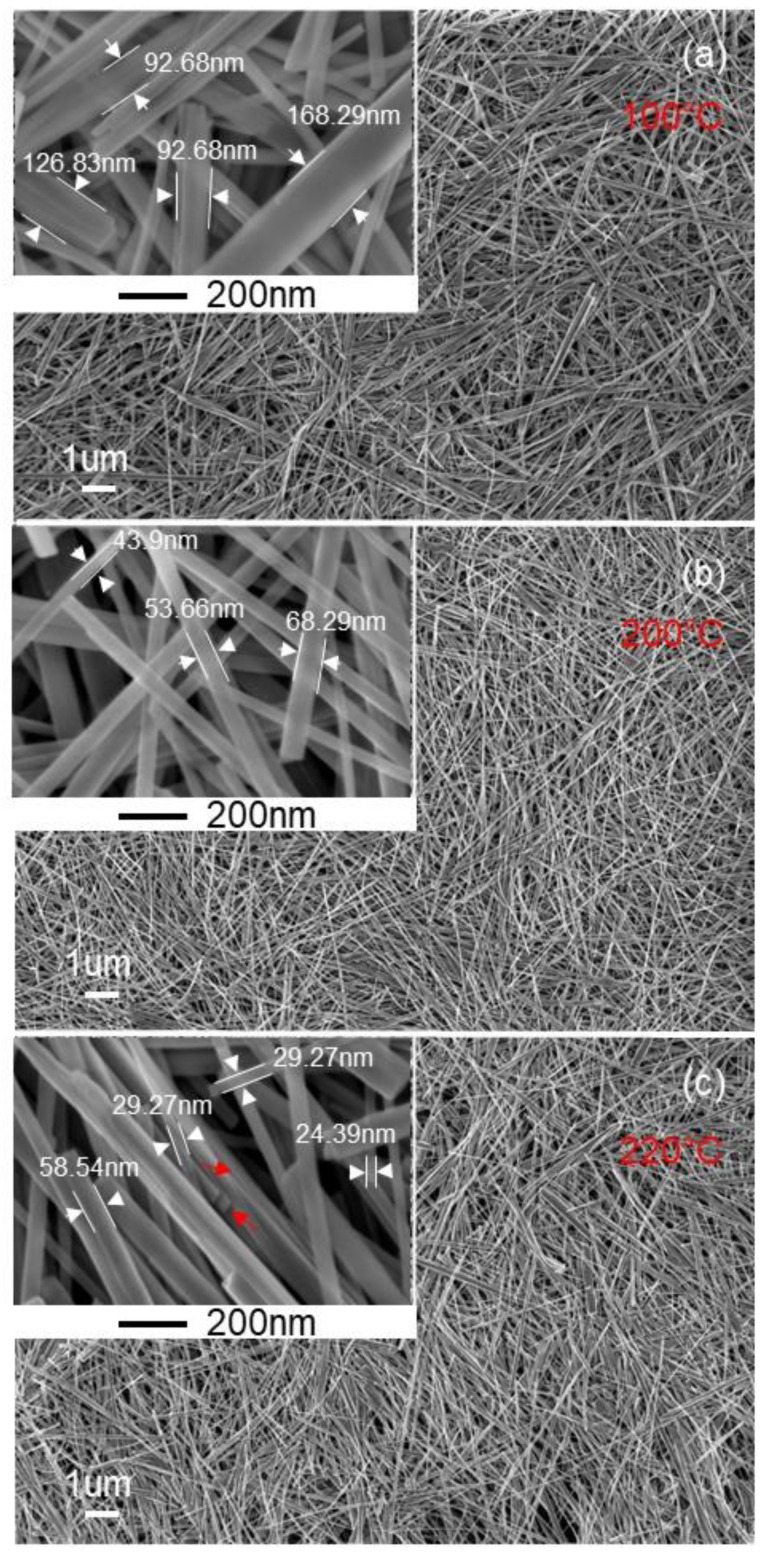
FEGSEM images of TiO_2_ nanobelts prepared at synthesis temperature of 200 °C and using TiO_2_ aggregate precursors of different sizes prepared at temperatures of (**a**) 100 °C, (**b**) 200 °C, and (**c**) 220 °C, respectively.

It can be observed that at the earlier stage (reaction time of 15 min) of the nanobelt growth process, coalesced nanoparticles coexist with nanosheet like particles, indicated by zones in Figure 8a,c. Nanoparticles were located at the nanotube edges (region 1 and 2 in Figure 8c), and beside this simple attachment, an alignment of coalesced nanoparticles takes place (region 3 in Figure 8c). In addition, the nanosheet shows both stretched and rolled structures. The indicated region 4 in Figure 8c shows the starting process of nanosheet rolling. However, all these steps are a consequence of different nanobelt growth stages, which will evolve in a later stage to a nanotube structure observed in Figure 9 and nanobelt structure shown in Figure 10. However, at closer inspection of the nanosheet structure at an earlier stage, with a synthesis time of 15 min, we find that it presents an assembly of nanoparticles, whose sizes range from 5 to 20 nm, as indicated in selected region of Figure 8a,c. This proves that these nanoparticles and aggregates are the primary building units for the nanosheet formation process. Furthermore, it is well accepted in the literature that the key point for the formation of nanobelt-like structure is the formation of sodium titanate nanobelt intermediate, in which the sodium ion (Na^+^) is inserted into space between TiO_6_ octahedra layers, balancing their negative charges [4,29]. From the present experiments, it can be inferred that the aggregate of TiO_2_ nanoparticles split up into nanosheets as a consequence of Na^+^ insertion and their rolling in a second stage to form nanotube in an intermediate stage. Typical TEM and HRTEM patterns of TiO_2_ nanotube are depicted in Figure 9, with similar structure of nanotube obtained using TiO_2_ nanoparticles in terms of asymmetrical walls. It can be seen that the nanotube exhibits four layers on one side and two layers on the other (Figure 9e), which indicates that the nanotubes are formed by the scrolling of several layers of nanosheet, as previously observed by other authors. The interplane on both sides is of 0.36 nm, which corresponds to the (010) crystallographic plane, and is the characteristic of monoclinic H_2_Ti_3_O7. It was reported for the same materials that the nanotube growth takes place along the (010) direction. Additionally, the interlayer distance between rolled nanosheets is about 0.76 nm closer to different reported values [29].

From XPS and EDX analysis in Figure 6 and Figure 7, it is clearly demonstrated that the sodium ions are incorporated in the TiO_2_ nanobelt, nanotube, and nanourchin, which suggest that it plays a role in their formation mechanisms. These observations indicate that nanobelts are formed by an orderly sticking of nanosheet and their coalescence in later stage; whereas nanourchins are formed by random assembly of the nanosheets.

The size dependence of the TiO_2_ nanobelt on the size of TiO_2_ aggregate precursor was demonstrated. Different sizes of TiO_2_ aggregate precursors were used to prepare TiO_2_ nanobelt, and the obtained results are depicted on Figure 10. It can be observed that the nanobelt length tends to increase with the increasing of the TiO_2_ aggregate precursor size. Additionally, the TiO_2_ nanobelt width increases from 50 to 200 nm (Figure 10), when the TiO_2_ aggregate precursor size increases from 50 to 200 nm (Figure 1). This confirms that TiO_2_ nanoparticles play a role in the formation of different observed morphologies. In fact, if we assume that the formation of observed morphologies goes through the TiO_2_ dissolution and precipitation, the TiO_2_ nanoparticles size will not have any effects on the final nanoparticle size. Additionally, the observation of TiO_2_ nanoparticles during the nanotube formation supports the mechanism through which sodium ions (Na^+^) induce exfoliation of TiO_2_ aggregates by insertion into the space between TiO_6_ octahedra layers and their coalescence to form nanosheets at later stage. Furthermore, the present results provide additional arguments to support some reported works in the literature and contradict others [30,32], in which it was claimed that during the hydrothermal synthesis process, TiO_2_ is dissolved through Ti–O–Ti bonds breaking and formation of sodium titanate nanosheet [29], which is converted to hydrogen titanate during the washing step and at a later stage to TiO_2_ nanobelt after the annealing process.

**Figure 11:**
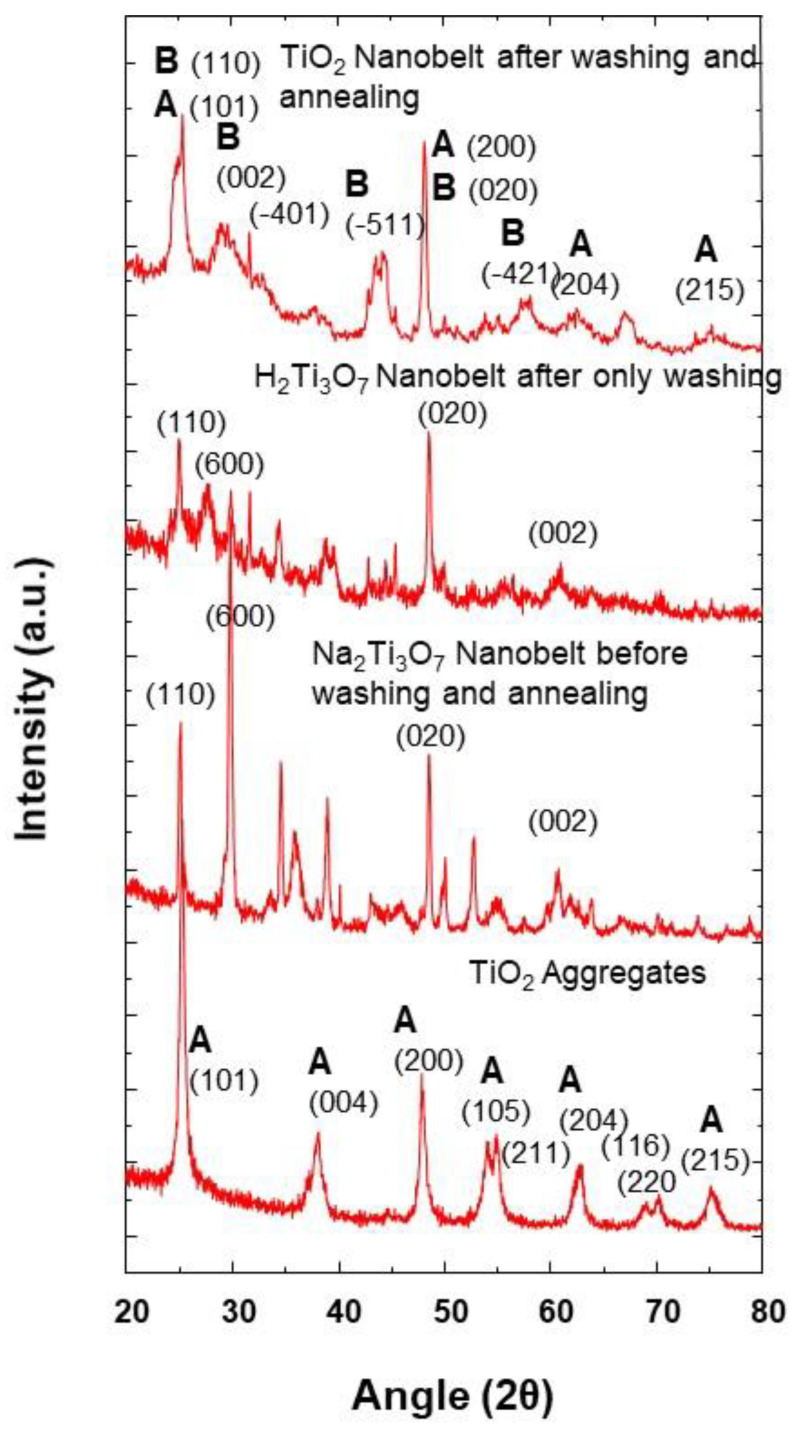
XRD pattern of TiO_2_ nanobelt at different synthesis stages as indicated and at the synthesis temperature of 100 °C (A: Anatase; B: TiO2-B).

It can be seen from the XRD results that the nanobelt powder, at different synthesis stages (Figure 11), shows a changing of crystalline structure. The TiO_2_ aggregates precursor is of anatase phase, with tetragonal structure, in which TiO_6_ octahedra are sharing their face and get stacked in a one-dimensional zigzag chain. During the synthesis of Na_2_Ti_3_O_7_ nanobelts, a crystalline transition takes place, and TiO_2_ anatase phase is transformed into an orthorhombic structure. In fact, the formation of sodium titanate nanobelt intermediate is obtained through the insertion of sodium ion (Na^+^) into the space between TiO_6_ octahedra layers, inducing the distortion of the initial structure.

From these XRD results obtained at the synthesis temperature of 100 °C, it can be inferred that the anatase TiO_2_ aggregate structure changes are a consequence of Na^+^ insertion and a strong repulsion between Na^+^ ions, which induces a distortion of the anatase crystalline structure. Similar behavior is observed with the insertion of Na+ ion in the case of Na ion batteries charging/discharging cycles [33]. However, after the washing step with hydrochloric acid solution, the H_2_Ti_3_O_7_ nanobelts are obtained as a consequence of proton exchange processes of sodium trititanate. From Figure 11, it can be seen that this exchanging of steps and the resulting orthorhombic structure of H_2_Ti_3_O_7_ (JCPDS Card No. 47-0124) are accompanied by some XRD peak modifications, in terms of the intensity enhancement of some peaks, and their decrease for some others [34,35]. These modifications indicate the distortion of the initial structure after ion exchanges. Additionally, after the annealing process and the removal of protons, a mixture of anatase (JCPDS 21-1272) and TiO_2_-B (JCPDS 35-0088) phases is obtained at the synthesis temperature of 100 °C. The obtained XRD pattern is similar to that obtained for the same mixture by Beuvier et al. [36]. A phase transition was observed when the morphology changed from nanotube to nanobelt, but with different compositions than those obtained at the synthesis temperature of 200 °C. It was reported by Zhang et al. that the TiO_2_ nanoparticle size has a strong impact on the phase transformation during the growth of coalesced nanoparticles [37]. In addition, the temperature also plays an important role in the phase transformation of TiO_2_ nanoparticles [26]. However, as when the temperature is changed the coalescence and/or growth of TiO_2_ nanoparticles take place, both the temperature and the size contribute to the phase transformation and a formation of different phase mixtures depending on the used synthesis temperature 100 and 200 °C. Furthermore, as it can be observed from Figure 4 and Figure 10, the peaks corresponding to the anatase phase are of lower intensity, which indicates that both of the latter synthesis temperatures produce a lower proportion of anatase, in agreement with different reported works in the literature [36]. During the synthesis process at a given temperature, the phase is also changed due to the insertion of different ions, and it is not necessary to dissolve and precipitate TiO_2_ octahedra. Furthermore, from these results, it is worth noting that the synthesis temperature plays a crucial role in the phase control of prepared nanobelt powders.

## 4. Conclusions

Different morphologies of TiO_2_ nanoparticles have been synthesized, in a large scale using hydrothermal synthesis technique and TiO_2_ aggregate as a precursor. Both nanotube, nanourchin-like, and nanobelt-like nanoparticles were obtained at low temperatures and over short times. Furthermore, it is demonstrated that a morphology control of prepared TiO_2_ powders could be achieved through the tuning of the synthesis temperature and time. The mechanisms formation of TiO_2_ nanobelt-like, nanourchin-like, and nanotube nanoparticles are illustrated to involve TiO_2_ nanoparticles coalescence and nanosheet intermediate, formed thanks to Na^+^ ions exfoliation. Furthermore, it was found that the prepared TiO_2_ nanomaterials properties were governed by those of TiO_2_ nanoparticles aggregate. It has been demonstrated that it is possible to tune the nanobelt size by using different TiO_2_ aggregates precursor sizes. Additionally, it was shown that the synthesis temperature enables the tuning of the phase’s composition of the nanobelt powders. The investigation of prepared powders performance, as anode material for Li-ion batteries, is under progress in our group.
